# An Exploration of Machine-Learning Estimation of Ground Reaction Force from Wearable Sensor Data

**DOI:** 10.3390/s20030740

**Published:** 2020-01-29

**Authors:** Danica Hendry, Ryan Leadbetter, Kristoffer McKee, Luke Hopper, Catherine Wild, Peter O’Sullivan, Leon Straker, Amity Campbell

**Affiliations:** 1 Curtin University, School of Physiotherapy and Exercise Science, Perth WA 6845, Australia; 2 Curtin University, School of Mechanical and Civil Engineering, Perth WA 6845, Australia; 3 Western Australian Academy of Performing Arts, Edith Cowan University, Perth WA 6050, Australia

**Keywords:** machine learning, inertial sensor, ballet, ground reaction force

## Abstract

This study aimed to develop a wearable sensor system, using machine-learning models, capable of accurately estimating peak ground reaction force (GRF) during ballet jumps in the field. Female dancers (n = 30) performed a series of bilateral and unilateral ballet jumps. Dancers wore six ActiGraph Link wearable sensors (100 Hz). Data were collected simultaneously from two AMTI force platforms and synchronised with the ActiGraph data. Due to sensor hardware malfunctions and synchronisation issues, a multistage approach to model development, using a reduced data set, was taken. Using data from the 14 dancers with complete multi-sensor synchronised data, the best single sensor was determined. Subsequently, the best single sensor model was refined and validated using all available data for that sensor (23 dancers). Root mean square error (RMSE) in body weight (BW) and correlation coefficients (r) were used to assess the GRF profile, and Bland–Altman plots were used to assess model peak GRF accuracy. The model based on sacrum data was the most accurate single sensor model (unilateral landings: RMSE = 0.24 BW, r = 0.95; bilateral landings: RMSE = 0.21 BW, r = 0.98) with the refined model still showing good accuracy (unilateral: RMSE = 0.42 BW, r = 0.80; bilateral: RMSE = 0.39 BW, r = 0.92). Machine-learning models applied to wearable sensor data can provide a field-based system for GRF estimation during ballet jumps.

## 1. Introduction

Ground reaction force (GRF) is a commonly measured biomechanical feature during impact-based activities such as landing from a jump [1,2,3,4,5]. Peak values of the GRF during jumping typically exceed several times an athlete’s body weight (BW) [4,5]. For example, laboratory-based studies have demonstrated that basketballers, volleyball players, and runners exhibit peak GRFs between 2–5 BW [4], and gymnasts land a frontsault with up to 15.8 BW [5]. Ballet dancers are aesthetic athletes who have been reported to perform up to 220 jumps within a single training session, from over half of which they land unilaterally [6], with peak GRFs commonly exceeding 4 BW [2,7]. High GRF during landings may increase the accumulated internal loads that these athletes experience during training, competition and performance, thus increasing susceptibility to musculoskeletal pain conditions [3,8]. For example, recreational athletes have demonstrated 3.4%–6.5% higher peak vertical GRF on landing when fatigued [3]. Similarly, high peak GRFs during impact-based activities have been associated with the development of lower limb musculoskeletal pain conditions [8]. Therefore, GRF is considered an important issue for dancers.

GRF is commonly measured in laboratory studies using force platforms [1,2,4,9,10]. The output from a force platform provides a complete GRF profile, allowing identification of the GRF at any point during the jump. However, force plates are expensive and restricted by their dimensions, and thus are typically unable to assess complicated athletic maneuvers, such as series of jumping tasks commonly performed in ballet. Importantly, these systems are not ecologically valid [11], i.e., they are unable to capture a dancer’s movement in a normal training environment or across a performance season or training period, where changes in movement due to factors such as fatigue may be common. As a result, there is a need for a field-based system for measuring GRF during jumping tasks. 

Recent advancements in wearable technology has opened the possibility of field-based GRF measurement, providing biomechanical insight in sports where laboratory-based measurement is challenging. For example, force insoles have been added to ski boots for analysis of ski jump landings [12], and bendable outsoles used for GRF measurement during walking [13]. Within a dance population, the addition of an insole or outsole to a ballet shoe is not possible due to the aesthetic and technical requirements of the athletic pursuit. Rather, wearable technology potential in this population lies in small, body-worn, commercially available wearable sensors [14,15,16,17,18,19]. 

Traditionally, wearable sensor accelerometer data has been used in the field during walking and running activities to estimate force directly using inverse dynamics [16,18,19,20]. However, given the noisy signal, this method has variable success [20]. Most current wearable sensors contain multiple hardware chips such as inertial measurement units (IMUs), which combine an accelerometer, magnetometer and gyroscope. Rather than directly entering the derived data into calculations, sports scientists are applying sophisticated machine-learning algorithms to indirectly estimate GRF using data from these sensors [14,15,17]. Machine-learning models have been applied to both multi-sensor and single sensor data in order to estimate GRF during running [14]. Using three IMUs, mounted on the sacrum and legs, Wouda et al. [14] demonstrated a root mean square error (RMSE) of 0.39BW (range = 0.21–1.25 BW), with a correlation coefficient of 0.96 across the GRF profile, and no significant difference in peak GRF between the predicted and gold standard force plate values. While these results are promising, running is characterised by a rhythmical, consistent and predictable movement profile. 

Machine learning has also been applied to estimate other biomechanical forces. For example, data from two IMUs for knee joint force estimation during sports-specific tasks, such as cutting and basic jumping tasks [21]. This model demonstrated reduced accuracy (average RMSE of 19.1%) compared to that used in running, potentially due to more variable movement patterns [21]. Further research is required for development of such models for GRF during complex, sports-specific tasks such as jumping. Additionally, within the unique context of dance, a system requiring a minimum number of sensors is required to conform within the aesthetic requirements.

The purpose of this study was to develop a series of machine-learning models capable of predicting peak GRF during bilateral and unilateral dance-specific jumping tasks. A field-based measurement of these biomechanical features would enable exploration of the role of GRF in the development of musculoskeletal pain conditions in people when engaging in lower-limb loading tasks. 

## 2. Materials and Methods

The Consensus-based Standards for the Selection of Health Measurement Instruments (COSMIN) provided guidelines for the design and reporting of this study [17]. 

### 2.1. Participants

Thirty female ballet dancers (mean (standard deviation, SD) age: 18.50 (1.68) years, mean (SD) weight (kg): 54.7 (3.3) kg) were recruited from ballet schools across Perth, Western Australia. Dancers were included in the study if they were aged 16 years or older and participating in a minimum of 6 hours of ballet training per week. Only female dancers were recruited for this study as the movement profile of females and males are different in ballet, and there is greater female participation at a pre-professional level. Both recreational and pre-professional dancers were included in the study to allow for greater diversity of skill level, and thus variability of movement for model development. Dancers were excluded from the study if they were currently injured or unwell. This study was approved by the university’s human research ethics committee (HRE2017-0185). Informed consent was obtained from all participants included in the study. 

### 2.2. Data Collection

Dancers attended a single data collection session at the university’s motion analysis laboratory. Following completion of a short questionnaire detailing their current dance participation and years of dance experience, body mass, height and limb measurements (lower limb length, knee width, ankle width) were recorded using calibrated scales (Tanita Corporation of America, Arlington Heights, IL, USA), a stadiometer (Mentone, Victoria, Australia) and a tape measure. 

All jumps were performed on a single force plate (Advanced Mechanical Technology, Inc., Water-town, MA, USA) operating at 2000 Hz. The force platform was covered with a thin, soft mat attached to the platform to better simulate a dance floor. 

**Figure 1:**
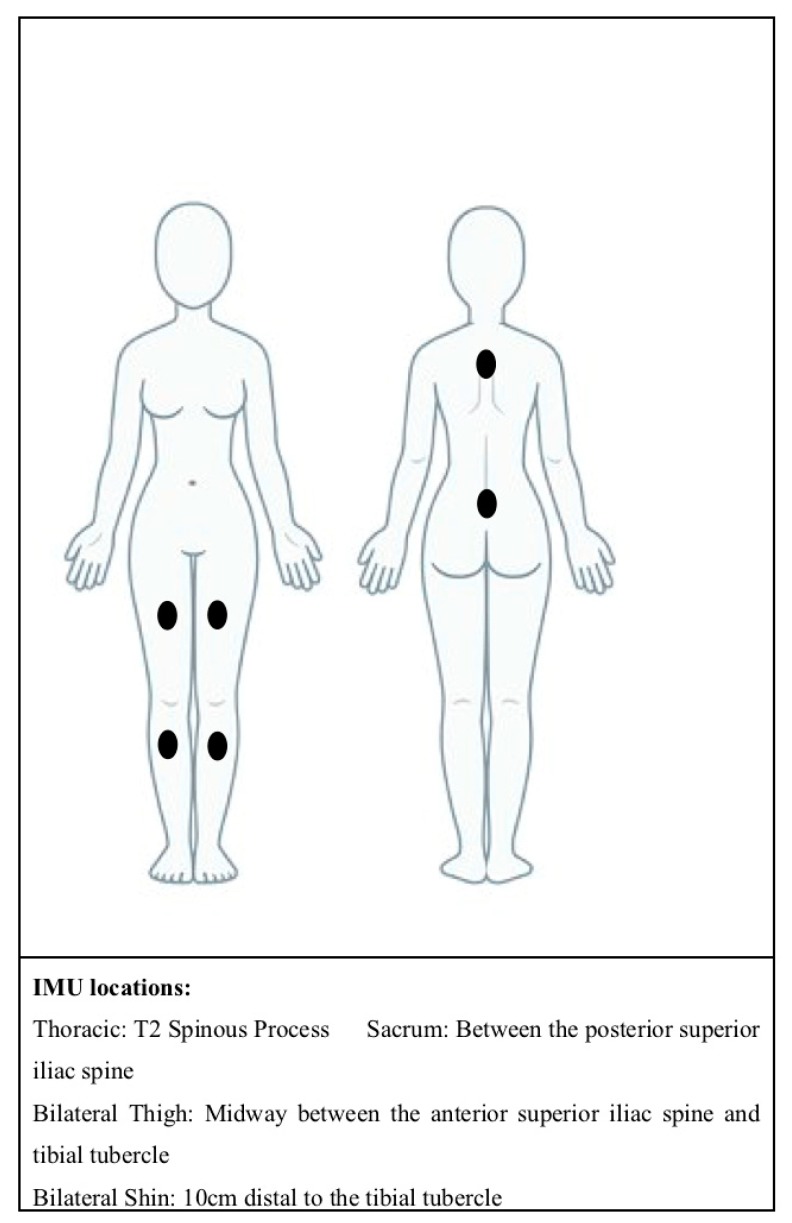
Anatomical locations of inertial measurement units (IMUs).

Dancers were fitted with six ActiGraph Link wearable sensors (ActiGraph Corporation, Pensacola, FL, USA), operating at 100 Hz and with the gyroscope and magnetometer enabled. The ActiGraph Link is a small commercially available tri-axial wearable IMU. The sampling frequency of 100 Hz was selected as this was the maximum sampling frequency available on this device. The ActiGraph sensors were secured to the skin using double-sided tape (3M 1522 Medical Tape, double sided, transparent, 3M, MN, USA), where the double-sided tape was placed between the sensor and the skin. This was then further secured using a single piece of hypoallergenic tape (Rocktape, Australia), which covered the sensor so that it did not dislodge during jumps. The double-sided tape is non-elastic and commonly used within biomechanical research, the hypoallergenic tape is elastic so as not to restrict the dancers’ movements. Sensors were placed on the thoracic spine, sacrum (recommended as this is close to an individual’s centre of mass) and bilateral shin and thigh (to capture lower limb movement) (See Figure 1). Lower limb sensors were placed anteriorly on the thigh to avoid obstruction of movement. All sensor locations also allowed for easy attachment to the dancer’s skin, reducing the potential impact of movement artefact from clothing interfering with the sensors. The sacrum sensor can be concealed easily, thus conforming with the aesthetic requirements of ballet. Data collection for each participant took approximately 45 min. 

### 2.3. Jumping Tasks

Following a self-directed warm up and sensor attachment, the dancers performed a series of bilateral and unilateral ballet specific jumps (Table A1). The tasks selected were performed in progressions that followed a typical ballet class format, i.e., jumps with bilateral landings, followed by jumps with unilateral landings. The number of repetitions of each task is presented in Table A1 and is also reflective of performance within a normal ballet class. All unilateral tasks were repeated on both lower limbs. 

### 2.4. Data Processing

Following data collection, ActiLife software (Version 6.13.3) was used to output date-time stamped files of each wearable sensor’s raw data: including tri-axial accelerometer, gyroscope and magnetometer outputs. Force platform data were down-sampled to 100 Hz to match IMU data. Both force plate and acceleration data were normalised to G-force (Gs). A customised LabVIEW program (National Instruments, Austin, TX, USA) was designed to allow semi-manual time synchronisation of wearable sensor data with force platform data. For this purpose, a single reviewer visualised the sum of the residuals of the sacrum sensor accelerometer data with the force plate data to align and check for synchronisation. Following time synchronisation, the program outputted a collated file of all wearable sensor and force platform data for each task.

### 2.5. Machine-Learning Model Development and Validation

While data were collected on 30 dancers, wearable sensor data from seven of the dancers had issues with hardware malfunctions. This was recognised when data was downloaded and visually inspected after data collection. Hardware malfunction issues included sensors not being accepted by the docking station to download data and sensors breaking during data collection and not collecting data. Therefore only 23 of the dancers had data that could be used in the development of the machine-learning model. Of these 23 dancers, 14 had data which were deemed adequately synchronised across all six sensors, allowing for exploration of multi-sensor models. Synchronisation issues were caused by a manufacturer fault in this brand of sensors, which can result in a between-sensor time shift. As a result, some sensors could not be synchronised due to large time differences between both the other sensors and the force platform. Adequate synchronisation of sensors was determined via visual observation of a single researcher, by alignment of the peaks of acceleration data, and matching the periods between these peaks.

**Figure 2:**
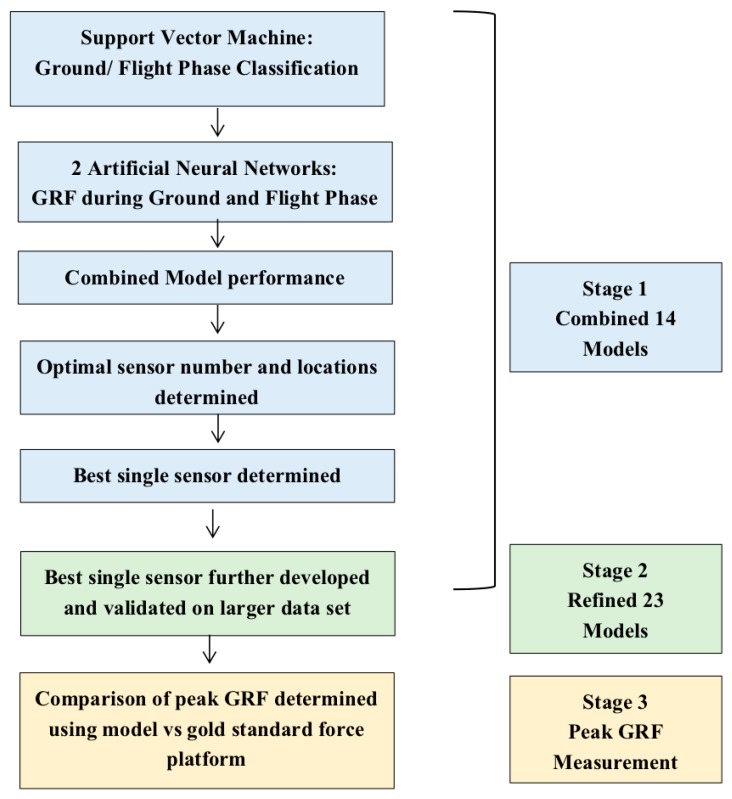
Flow chart demonstrating model development and validation process and model architecture.

Visual inspection of the data revealed that the magnetometer raw data was unstable and not representative of the dancers’ movement, thus this data was not utilised. Only the accelerometer outputs were used in the development of the models. Gyroscope data was not used in the development of the model to avoid having too much data that was similar to each other, where acceleration is related to velocity, and is also more closely related to force. The model was developed in a number of stages, with the final goal being to achieve a model capable of estimating peak GRF. The stages of development are demonstrated in Figure 2, and described below (Section 2.5.1, Section 2.5.2 and Section 2.5.3)

#### 2.5.1. Stage One: Initial Model Development and Evaluation

**Figure 3:**
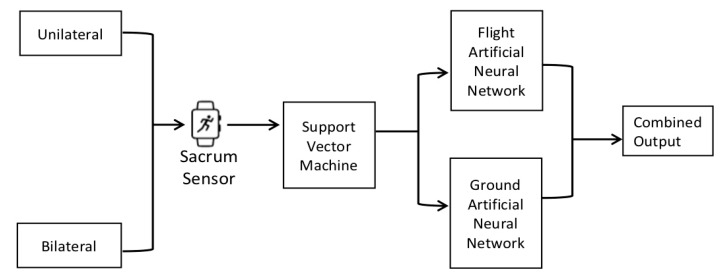
Model architecture.

Based on initial experimentation, two pilot model designs were developed using data from 14 of the dancers; one for unilateral landings, and one for bilateral landings. The models were initially trained on 12 dancers (training set) and evaluated on the remaining two (test set). Model architecture is shown in Figure 3. The models incorporated a support vector machine (SVM) for flight and ground phase classification with separate artificial neural networks (ANN) for the GRF estimation during each phase. The models were constructed so that the final output model only required single data points and no historic points, thus GRF could be predicted for each data point individually, allowing for the potential of real-time GRF estimation.

##### Support Vector Machine to Classify Ground and Flight Phases

The SVM was developed using a gaussian kernel function, to determine if a data point was classified within the flight or ground phase of the jump. The input for the SVM was the vector magnitude of the acceleration data from the IMUs, measured in Gs at 100 Hz for the period of the activity. During the ground phase, the segment accelerations were coupled to the GRF, whereas during the flight phase the GRF determined by the force platform is reduced to zero, while the segment accelerations are not. Segment accelerations refer to the acceleration vector of segments of the body such as the torso, thigh or shin. Therefore, a data point was assigned a ground phase label if the GRF recorded by the force plate was greater than 0.05 BW, and assigned a flight phase label if it was less than 0.05 BW. 

An equal number of data points for every type of jump performed by each dancer were sequentially arranged, before being rearranged randomly using the MATLAB Random Number Generator (MathWorks, Inc., Natick, MA, USA), to produce an overall training set. As the data was collected at 100 Hz, a data point is defined as a time period of data that is 1/100th of a second in duration. The first 500 data points from the overall training set were taken to train the SVM, with a five-fold cross validation process used, allowing for selection of the best-performing model with a smaller training set. The first 200 data points from the test set were then used to assess the performance of the SVM. A reduced sample was decided upon due to the reduced data requirements of a SVM, requiring smaller training and test data, and to prevent the occurrence of overfitting. Additionally, the smaller test set was used to enable more efficient training and testing of the models, given the large number of models being developed. Overfitting is when a model corresponds too closely or exactly to a particular data set and, therefore, may not be able to predict future observations reliably. Within the context of machine learning for wearable sensors and human movement, this can occur due to a data set which does not provide sufficient variability of movement (i.e., is trained on a set of data that is all very similar, thus the model learns only to recognise these patterns) [22].

To evaluate SVM classification accuracy for each possible sensor combination, confusion matrices were constructed using the percentage of data points that were correctly predicted, for both unilateral and bilateral jumps. 

##### Artificial Neural Networks (ANNs) to Estimate Ground Reaction Force (GRF) During Ground and Flight Phase

Separate ANNs were developed; one for the ground phase and one for the flight phase of the jump. Optimal ANN architecture was determined using an iterative loop, to determine which number of neurons in each hidden layer resulted in most accuracy when all six sensors were used. For the flight phase, only one hidden layer was assessed, and for the ground phase both single and double hidden-layer networks were investigated. Single and double hidden-layer networks with a lower bound of one and an upper bound of 35 in each layer were explored when determining the hyperparameters. All models were trained starting with randomly generated weights.

##### Combined 14 Models to Estimate GRF Across Whole Jump Activity

The SVM and two ANNs were combined in two models, one for bilateral landings and one for unilateral landings. Separate models were used for each type of landing to improve accuracy due to the differences between bilateral and unilateral landings. In each individual model, once a data point was classified by the SVM as being within the ground phase or the flight phase of the jump, it was fed into the corresponding neural network, as demonstrated in previous reporting by Leporace et al. [17]. This structure allowed for each individual data point to be introduced to the machine-learning model to produce an estimation of GRF profile across the whole activity. The model architecture is shown in Figure 3.

To evaluate the combined model, incorporating the SVM and both ground- and flight-phase ANNs, the GRF estimations across the total GRF curve were compared with force platform ‘gold standard’ GRF using RMSE, as well as Pearson’s correlation coefficients to provide indication of standardised fit. The total GRF curve of each jump was considered including both the flight phase and subsequent ground phase. 

##### Determination of Optimal Sensor Number and Locations

The performance of all sensor combinations was compared by utilising a SVM, an ANN and the Combined 14 Models for each sensor combination. For both unilateral and bilateral jumps, 63 models were developed, one for each different combination of sensors (all six sensors, all combinations of five sensors, all combinations of four sensors, etc.). SVM performance was evaluated using a confusion matrix for classification accuracy. ANN and Combined 14 Model performance was evaluated by comparison with force platform GRF across the whole jump activity using RMSE and Pearson’s correlation coefficients. This was determined using a leave-two-out-cross validation approach, where the model was trained on 12 dancers and evaluated on the remaining two, and this was iteratively repeated on all combinations of two dancers (total of 91 combinations, yielding a total of 11,466 models trained and tested (63 [possible sensor combinations] × 91 [combinations of dancers] × 2 [unilateral/bilateral]). The 10 best possible combinations (number and locations) of sensors were saved. The leave-two-out cross validation approach was used to allow greater generalisability of the model given the smaller sample size. The best single-sensor model based on location was identified for both unilateral and bilateral jumps. The best single-sensor model was determined by looking at the SVM, ANN and Combined Model results together and determining which single-sensor location performed with greatest accuracy. Additionally, one of the top performing models from the leave-two-out-cross validation for this single sensor was selected to be integrated into a user-friendly program to use for stage three of this development.

#### 2.5.2. Stage Two: Refinement and Evaluation of Single Sensor Models Using a Larger Sample

Single sacral-sensor models for both the bilateral and unilateral jumps were refined using data from 23 dancers. The model was developed using a leave-one-out-cross validation where it was iteratively trained on 22 dancers’ data and evaluated for the remaining one (total of 23 combinations) [13]. 

To evaluate the performance of the Refined 23 Models, the average RMSE and correlation coefficients were determined for the GRF profile across the jump activity in comparison with the gold standard force platform GRF profile. One of the top performing models was selected to be integrated into a user-friendly program to use for stage three of this development.

#### 2.5.3. Stage Three: Validation of Combined 14 Models and Refined 23 Models to Determine Peak GRF Using Single Sensor

For both the 14 dancer and 23 dancer single-sensor models, one of the top-performing models was selected to be integrated into a user-friendly MATLAB (MathWorks, Inc., MA, USA) program to use for peak GRF output (maximum value within the ground phase) for a selection of trials for each of the 23 participants. Bland–Altman plots were constructed to determine the level of agreement between the machine-learning models and the gold standard force platform peak GRF values.

## 3. Results

### 3.1. Stage One: Support Vector Machine, Artificial Neural Network and Combined 14 Models Performance

The performance of the SVM when all six sensors were used demonstrated an average 87.8% degree of accuracy for unilateral jumps and 80.8% for bilateral jumps. Using all 6 sensors, the Combined 14 Models, trained and tested on 91 combinations of dancers, demonstrated an average RMSE of 0.24 BW for unilateral landings and 0.21BW for bilateral landings, with average correlation coefficients of 0.96 and 0.98, respectively.

### 3.2. Stage One: Determination of Optimal Sensor Number and Locations

**Figure 4:**
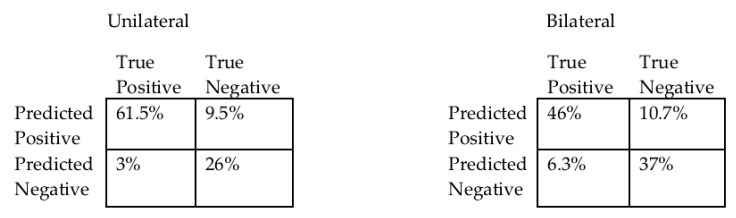
Confusion matrices for support vector machine performance with single sacrum sensor.

The performance of the stage-one SVMs tended to improve with fewer sensor inputs. This is demonstrated in Table 1 which shows the best sensor location combinations for one to five sensors. The sacral sensor had the highest accuracy of any single sensor. Confusion matrices for the single sacral sensor are demonstrated in Figure 4.

The performance of the top 10 performing sensor combination Stage one ANNs and Combined 14 Models is shown in Table 2. 

### 3.3. Stage One: Combined 14, Best Single-Sensor Models

**Figure 5:**
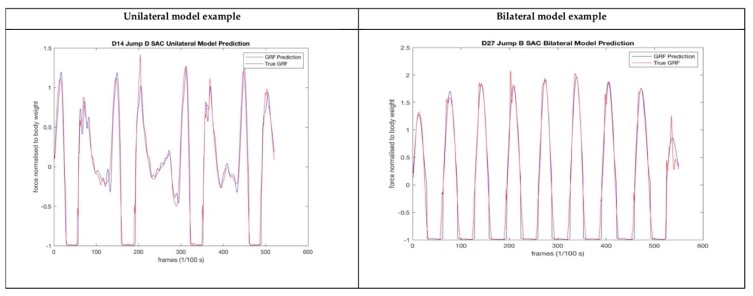
Outputs from unilateral and bilateral models—ground reaction force (GRF) profiles.

Considering the performance of the model overall, it was determined that the best single-sensor model was the sacrum sensor, with an RMSE of 0.25 BW for unilateral landings and 0.24 BW for bilateral landings, with a correlation coefficient of 0.95 and 0.98, respectively. Considering both the SVM and the Combined 14 Model results this was also considered the best sensor combination overall. Examples of the GRF profile output by the force plate and the best single sensor model are shown in Figure 5.

### 3.4. Stage Two: Refined 23 Models

The accuracy of the Refined 23 Models’ capability to estimate the GRF profile, accounting for all 23 dancers’ data, is demonstrated in Table 3. 

### 3.5. Stage Three: Combined 14 Models and Refined 23 Models’ Ability to Determine Peak GRF

**Figure 6:**
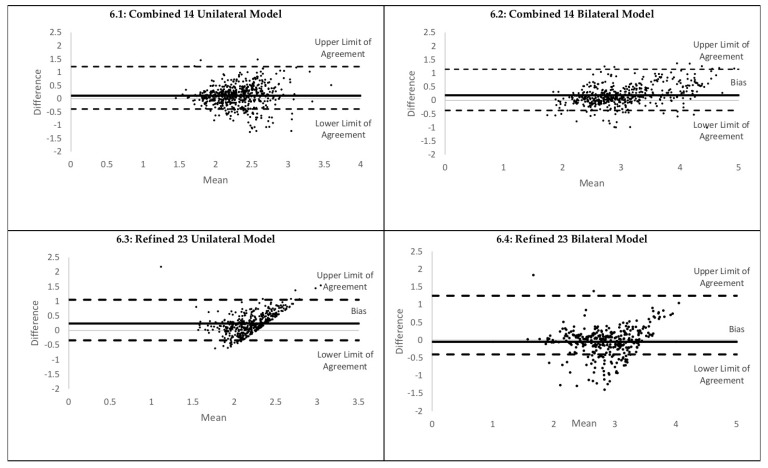
Bland–Altman plots for peak GRF estimation performance.

The best bilateral and unilateral model determined for the Combined 14 Models and Refined 23 Models was evaluated. The mean (SD) peak GRF as determined by the force platform was 2.35 BW (0.38) for the unilateral jumps and 3.13 BW (0.72) for the bilateral jumps. The mean (SD) peak GRF for the Combined 14 Models was 2.24BW (0.35) for the unilateral model and 2.95 BW (0.58) for the bilateral model. For the Refined 23 Models the mean (SD) peak GRFs were 2.12 (0.20) and 3.28 BW (0.62), respectively. The Bland–Altman plots are demonstrated in Figure 6. 

## 4. Discussion

The overall aim of this study was to validate the estimation of peak GRF from wearable sensor data during dance jumping tasks against gold standard force plate data. This aim was achieved through a multistage approach to development. The model architecture was developed within the first stages using 14 dancers, and evaluation of the different sensor numbers and locations determined that a single sacrum-mounted sensor performed with the same accuracy as the multi-sensor models for both unilateral and bilateral jumps. Interestingly, the second-stage model, developed on a larger sample, yielded poorer accuracy. 

Regardless of the number and locations of sensors, all developed models in stage one performed well. All of the top 10 sensor combinations for the Combined 14 Models demonstrated an RMSE of less than 0.35 BW for the unilateral models and 0.24 BW for the bilateral models. This model performance was superior to previous machine-learning model developments for GRF using data from three sensors on eight participants to predict GRFs during running (average RMSE of 0.40 BW) [14]. Additionally, the accuracy demonstrated in the current was similar to that shown for a knee joint reaction force machine-learning model, developed on data from 13 participants [21]. Their model achieved an average RMSE of 16.7% for unilateral jump landings and 25.9% for bilateral. Table 4 demonstrates a tabulated comparison of the results of the existing study compared with previous reporting. Additionally, the single sacrum sensor Combined 14 Models and Refined 23 Models were capable of detecting peak GRF with a similar mean difference between the model and the gold standard force platform. For the single sacrum sensor unilateral Combined 14 Models the mean difference was 0.11 BW and for the single sacrum sensor bilateral Combined 14 Models the mean difference was 0.19 BW. Similarly, for the single sacrum sensor unilateral Refined 23 Models the mean difference was 0.22 BW and for the single-sacrum sensor bilateral Refined 23 Models it was 0.18 BW. These mean differences were slightly higher than that demonstrated by Wouda et al, where the peak GRF mean difference demonstrated between their model and the force platform was 0.10 BW [14]. Overall the current study’s findings suggest that the application of a machine learning approach to wearable sensor dancer for GRF estimation during complex athletic jumping activities, provides an accurate means to field-based estimation of GRF.

The Combined 14 Models development revealed the most accurate number of sensors and sensor locations for the unilateral model consisted of five sensors, and the second most accurate of all six. Interestingly, the single sacrum sensor was almost as accurate as a combination of multiple sensors, with the same RMSE, of 0.25 BW as these multi-sensor combinations. For the sacrum bilateral model, the difference between the best performing multi-sensor combination (sacrum and thoracic sensor) was only 0.04 BW. Additionally, regardless of whether a multi-sensor or single-sensor model was used, there was excellent correlation between the machine learning models and gold standard force platform (0.95 and 0.96, respectively). A similar difference existed for the bilateral model, which displayed stronger correlation than the unilateral. This was unexpected, as previous literature has suggested that for machine learning applied to wearable sensors and human movement, multiple sensors are advisable, as it can provide the highest recognition rate [23]. The results of the SVM suggest that, within the current study, the use of more sensor locations resulted in poorer classification of ground or flight phase, thus effecting the rest of the model. To date, no other researchers have demonstrated the use of machine learning with a single sensor; only one other study has utilised a single sensor machine learning model for GRF estimation during sidestepping and running [15]. There are multiple practical benefits of using a single sensor as opposed to multiple sensors; a single sensor is more affordable, has a reduced athlete and analysis burden, and does not require synchronisation with other sensors, thus reducing overall processing demands. 

The best single-sensor location was the sacrum. This was of interest as, currently within the sporting environment the most common location for a single sensor appears to be on the upper back [24,25,26,27]. For example, when sensors are used in team sports for quantification of training volumes and impacts, as part of athlete monitoring regimes, the sensor is most commonly mounted to the upper back [25,27]. While the single thoracic sensor still featured within the top 10 performing sensor combinations in the unilateral model, as found during feature extraction, it had a 0.10 BW higher RMSE than the sacrum sensor, thus was less accurate. Interestingly, the RMSE was not different between sacrum and thoracic mounted sensors in the bilateral model. One other machine-learning study has demonstrated the use of a single sacrum sensor, showing an error of up to 29.7% during running, which would equate to approximately 0.7 BW, given that during running the GRF attained can be up to 2.5 BW [15]. Thus, the models developed in the present study performed with greater accuracy. A sacrum-mounted sensor is also the most feasible sensor location for the application to dance, conforming to both aesthetic and movement requirements. Within other sports, the results of our study suggest that if sports scientists would like to objectively quantify impact loading, particularly for single limb loading activities as part of athlete monitoring, a sacrum mounted wearable sensor may be more accurate when compared to an upper back-mounted sensor.

**Figure A1:**
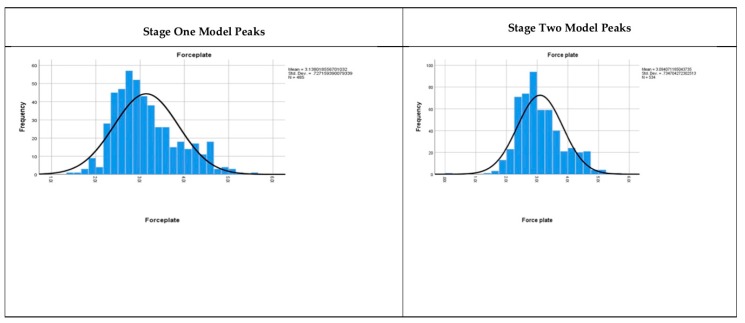
Frequency Histograms Representing Data Distribution.

When the single sensor sacrum model was further developed in the Refined 23 Models, the mean RMSE increased to 0.42 BW for unilateral jumps and 0.39 BW for bilateral jumps. Additionally, the correlation coefficients also reduced to 0.80 for the unilateral model and 0.92 for the bilateral model. While in theory, a larger data set should improve generalisability of the model and model performance, it is likely that the reduced accuracy of the models seen in the Refined 23 Models is due to overfitting. When the models were trained with the larger data set, the data set was skewed. This is common of normal human movement and one of the common challenges within machine learning [22], where in this case the dancers demonstrated a small number of variable GRF profiles. This was clearly highlighted in the peak GRFs Bland–Altman plots. When peak GRF was output using the Refined 23 Models, the unilateral model did not demonstrate peaks greater than 2.29 BW and the bilateral 4.01 BW, despite the gold standard force platform demonstrating greater values up to 3.85 BW and 5.51 BW for unilateral and bilateral jumps respectively. This cropping of values in the Refined 23 Models was not evident in the Combined 14 Models development. This has not been reported before and, given that increased data have been reported to increase accuracy, is surprising [22]. Further exploration of the data set using frequency histograms for the bilateral jumps (see Figure A1), demonstrating the range of GRFs used for training the models in the development of the Combined 14 and Refined 23 Models confirmed this hypothesis. The dancers landed with reasonably consistent GRFs, with the majority of peaks falling between 1.5–2.5 BW for unilateral jumps and 2.0–2.8 BW for bilateral jumps. Furthermore, the Refined 23 Models’ data shown in Figure A1 appears to be skewed towards smaller GRFs. This was likely due to the nature of the jumping tasks that were utilised, and represented an imbalanced data set [22]. Future research aiming to determine peak GRF during athletic tasks could potentially firstly endeavour to train the model with a large range of peak GRFs and also train the model specifically to detect the peak as opposed to the whole curve. 

### Strengths and Limitations

The models developed in this study can be used to estimate the GRF during impact-based activities in the athletic area of dance. While the authors acknowledge that dance is a niche athletic area, this study provides a proof of concept that could be easily applied to other sports, thus is highly translatable. The accuracy achieved is promising with a number of strengths. The models were developed using a relatively large sample compared to other studies, and additionally this sample included a range of dance ability thus increasing generalisability of the models. This study was limited to estimation using only IMU acceleration outputs. While the use of only accelerometer potentially reduces processing time and promotes longer battery life in the sensor, it only allows for resultant ground reaction force estimation with no indication of the direction of the forces. Future developments of machine-learning algorithms should consider utilising well calibrated magnetometer and gyroscope data to allow for force direction. Furthermore, by accurately estimating the GRF combined with specific segments kinematics, traditional inverse dynamics models could be applied to potentially calculate external joint forces at every joint. Additionally, the ActiGraph Link sensor used in this research was limited to a maximum sampling frequency of 100 Hz. A higher sampling frequency may provide more accurate results but also creates a greater burden of analysis. 

Despite the very strong correlations and low RMSE reported for the full GRF profile, the Refined 23 Models demonstrated an overfitting error that led to reduced accuracy in estimation of large peak GRF values during jumping. This suggests that future machine-learning endeavours on athletic pursuits with large variability need to manage data carefully to ensure it encompasses the full variety of movement and is normally distributed. Finally, the models developed through the different stages of this research used different validation techniques dependent on the sample size presented for the model. Further research evaluating the most beneficial validation of machine learning models based on sample sizes is needed. Finally, the sensors may not always represent movement of true ‘rigid segments’ as they are fixed to soft tissue and may come loose. This risk was minimised by attaching the sensor with tape, participants wearing fitted clothing and securing clothing away from the sensor where possible to minimise movement artefact.

## 5. Conclusions

The current study demonstrates that the novel application of machine learning to wearable sensor data allows for accurate estimation of peak GRF and the GRF profile during dance-specific jumping tasks. Interestingly, feature extraction testing revealed that a single sensor was capable of predicting GRF with the same degree of accuracy as a multi-sensor model. No previous reports have demonstrated the use of machine learning applied to a single wearable sensor on a sample of this size and with the degree of accuracy shown in this study. 

While the results are promising, the development did come with challenges. When the model was trained and tested on a larger sample, the accuracy of the model deteriorated and there appeared to be overfitting of the model, resulting in a cropping of peak forces. This is reflective of an imbalanced data set which is considered typical to normal human movement, and movement that is performed by a highly trained, aesthetic population. Additionally, challenges of hardware malfunctions and synchronisation problems reduced the overall data set that was available for model development. 

These results provide scope for the use of a single wearable sensor, combined with machine learning, to accurately estimate near real-time GRF within a dancer’s normal training environment. While developed within the niche athletic area of dance, the models developed in this research demonstrate the feasibility of this approach, which could be applied to other lower limb-loading sports and activities, providing a field-based measurement system for biomechanical quantification. This system, and future developments of it, could be used for athlete monitoring, both clinically and in research settings, for the provision of field-based objective quantification of GRF’s during training, competition and performance could lead to an improved understanding of musculoskeletal pain conditions.

## Figures and Tables

**Table 1:** Support vector machine (SVM) performance for best sensor location combinations for each number of sensors.

# Sensors	Unilateral		Bilateral	
	Best Combination	% Correctly Predicted	Best Combination	% Correctly Predicted
1	Sx	89.3	Sx	83.6
2	Sx, LSh	88.5	Sx, Tx	82.8
3	Sx, Tx, RSh	88.3	Sx, LTh, RTh	78.5
4	Sx, Tx, LSh, RSh	86.3	Sx, Tx, LTh, RTh	82.3
5	Sx, Tx, RTh, LSh, RSh	88.5	Sx, Tx, LTh, RTh, RSh	76.5

Key: Sx- Sacrum, Tx- Thoracic, LTh- Left Thigh, RTh- Right Thigh, LSh- Left Shin, RSh- Right Shin.

**Table 2:** Artificial neural network (ANN) and Combined 14 Model performance of top 10 performing unilateral and bilateral jump models ranked by degree of accuracy from most to least accurate.

Sensor Combinations	Flight Phase (ANN1) RMSE (BW) Mean	Ground Phase (ANN2) RMSE (BW) Mean	Combined (Flight and Ground Phase) RMSE (BW) Mean	Correlation Coefficient Mean
**Unilateral**				
Sx, Tx, LTh, RTh, LSh	0.05	0.27	0.24	0.96
ALL	0.05	0.28	0.25	0.96
Sx, Tx, LTh, RTh	0.05	0.28	0.25	0.96
Sx, Tx, LTh	0.05	0.28	0.25	0.96
Sx, Tx	0.05	0.28	0.25	0.96
Sx, LSh, RSh	0.05	0.28	0.25	0.96
Sx, LTh, RTh, LSh, RSh	0.05	0.28	0.25	0.95
Sx, LTh, RTh	0.05	0.28	0.25	0.95
Sx	0.05	0.29	0.25	0.95
Tx	0.05	0.40	0.35	0.90
**Bilateral**				
Sx, Tx	0.04	0.26	0.20	0.99
Sx, Tx, LTh, RTh, LSh	0.04	0.26	0.20	0.99
Sx, Tx, LTh	0.04	0.27	0.21	0.98
All	0.04	0.27	0.21	0.98
Sx, Tx, LTh, RTh	0.05	0.29	0.22	0.98
Tx, LTh, RTh, LSh, RSh	0.04	0.31	0.24	0.98
Tx, RTh, LSh	0.04	0.31	0.24	0.98
Sx, LTh, RTh	0.04	0.31	0.24	0.98
Sx	0.04	0.32	0.24	0.98
Tx	0.04	0.31	0.24	0.98

Key: Sx- Sacrum, Tx- Thoracic, LTh- Left Thigh, RTh- Right Thigh, LSh- Left Shin, RSh- Right Shin. ANN1- Flight Artificial Neural Network, ANN2- Ground Artificial Neural Network, RMSE- Root Mean Square Error, BW- Body Weight.

**Table 3:** Accuracy of final model estimation of GRF across complete curve.

Model	SVM to Identify Flight or Ground Phase Accuracy (%) Mean (Range)	Flight Phase (ANN1) RMSE (BW) Mean (Range)	Ground Phase (ANN2) RMSE (BW) Mean (Range)	Combined (Flight and Ground Phase) RMSE (BW) Mean (Range)	Correlation Coefficient Mean (Range)
**Unilateral**	83.17 (69.93–92.66)	0.05 (0.03–0.06)	0.30 (0.19–0.46)	0.42 (0.22–0.61)	0.80 (0.55–0.97)
**Bilateral**	84.06 (75.40–95.59)	0.04 (0.02–0.05)	0.27 (0.18–0.53)	0.39 (0.25–0.67)	0.92 (0.71–0.98)

**Table 4:** The results of the current study compared with results of previous reports for application of machine learning to wearable sensor data for GRF estimation.

Reference	Participants Used for Development	Number of Sensors	Sensor Locations	Machine Learning Approach	Movement Tasks	Variable Measured by Machine Learning Approach	Average RMSE
Current Study	23 female dancers (Stage one developed on 14 dancers, stage two on 23)	All combinations of six, five, four, three, two and one sensors. Demonstrated a single sensor approach in final reporting	Bilateral thigh, bilateral tibia, sacrum, thoracic	SVM and ANN	Unilateral and bilateral jumps	Resultant GRF across all data points of GRF profile, peak GRF.	Stage one development: Unilateral: 0.25 BW Bilateral: 0.24 BW Stage two development: Unilateral: 0.42 BW Bilateral 0.39 BW
Wouda et al., 2018 [14]	Eight runners	Three sensors	Bilateral leg, sacrum	ANN	Running	Vertical GRF across all data points of GRF profile, peak GRF	0.40 BW
Johnson et al., 2019 [15]	Did not specify	One sensor	Sacrum	Convolutional Neural Network	Running and side stepping	Three-dimensional GRF across all data points of GRF profile	19.7% (sidestep)–29.7% (run) of BW
Stetter et al., 2019 [21]	13	Two sensors	Thigh and shin	ANN	Running, running with turn, sprint start, full stop, side cutting maneuvers, walking, walking with turning, unilateral and bilateral jumping and landing	Three-dimensional knee joint reaction force	Vertical: 19.1% of BW Anterior/ Posterior: 21.8% of BW Medial/Lateral: 38% of BW

Abbreviations: SVM- Support Vector Machine, ANN- Artificial Neural Network, GRF- Ground reaction force, BW- Body weight.

## Appendix A

**Table A1:** Description of Tasks.

Name.	Description	Image Demonstrating Movement
Bilateral Landings		
Sauté in first position	The dancer commences in first position of the feet (lower limbs externally rotated and heels placed together) and performs 8 bilateral vertical jumps landing bilaterally.	
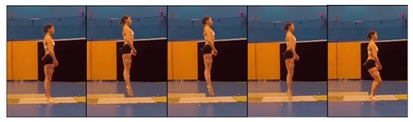
Changement in 5th position	The dancer commences in fifth position of the feet (lower limbs externally rotated and feet crossed) and performs 8 vertical jumps changing the front foot upon landing.	
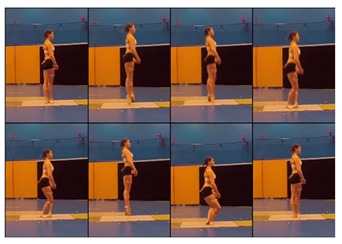
Entrechat Quatre	The dancer commences in fifth position of the feet (lower limbs externally rotated and feet crossed) and performs 4 vertical jumps beating the legs in air before landing bilaterally with the same foot in front. This was performed with the right leg and left leg starting in front.	
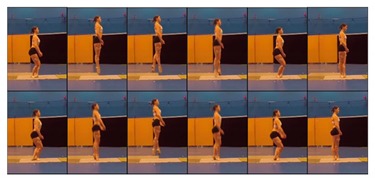
Unilateral Landings		
Assemblé	The dancer commences in 5th position and swishes one leg out to the side as they take off, they gather the legs in the air together and land before immediately taking off for the next jump.	
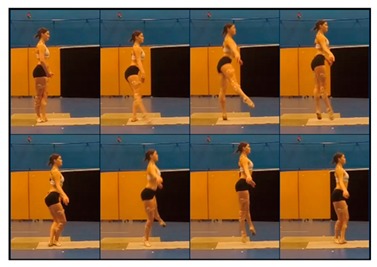
Jeté ordinaire	The dancer commences in 5th position and swishes one leg out to the side as they take off, they then land on the limb that they swished to the side.	
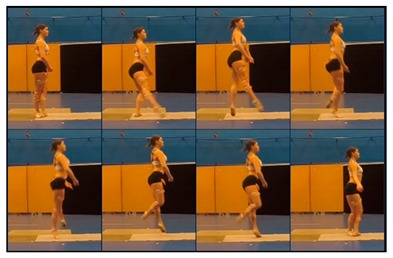
Temps levée	A single leg vertical jump and land performed 5 times in succession.	
